# Case report: The utilization of crizotinib and brentuximab vedotin as a bridge to autologous stem cell transplantation and followed by CD30-directed CAR-T cell therapy in relapsed/refractory ALK+ ALCL

**DOI:** 10.3389/fimmu.2024.1346001

**Published:** 2024-02-05

**Authors:** Wanying Liu, Jiaying Wu, Xi Ming, Qi Zhang, Delian Zhou, Rubing Zheng, Mi Zhou, Zhen Shang, Liting Chen, Xiaojian Zhu, Yi Xiao

**Affiliations:** Department of Hematology, Tongji Hospital, Tongji Medical College, Huazhong University of Science and Technology, Wuhan, Hubei, China

**Keywords:** anaplastic large cell lymphoma, T-cell non-Hodgkin lymphoma, crizotinib, CAR T cell therapy, autologous stem cell transplantation

## Abstract

**Background:**

Anaplastic lymphoma kinase-positive anaplastic large cell lymphoma (ALK+ ALCL) is a rare, mature T-cell non-Hodgkin lymphoma. The prognosis of patients with relapsed or refractory ALCL following first-line chemotherapy is extremely poor. NCCN guidelines recommend intensified chemotherapy with or without ASCT consolidation for r/r ALCL, however, this is not an effective treatment for all ALK+ALCL.

**Case report:**

Herein, we report a patient with relapsed/refractory ALK+ ALCL who received crizotinib and brentuximab vedotin as bridging therapy, followed by autologous stem cell transplantation and sequential anti-CD30 CAR T cell therapy.

**Conclusion:**

The patient achieved complete remission and long-term disease-free survival of months and continues to be followed up. The combination therapy model in this case may provide guidance for the management of relapsed/refractory ALK+ ALCL, and further prospective trials are needed to confirm its effectiveness.

## 1 Introduction

Anaplastic lymphoma kinase-positive anaplastic large cell lymphoma (ALK+ ALCL) is a rare aggressive systemic T-cell non-Hodgkin’s lymphoma (NHL), contributing approximately 6–7% of mature T-cell lymphomas. In 2016, the World Health Organization (WHO) classified anaplastic large cell lymphoma into four categories: ALK+ ALCL, ALK-negative ALCL (ALK− ALCL), primary cutaneous ALCL, and breast-implant-associated ALCL (BIA-ALCL). ALK+ ALCL is more common in children and young adults, with a male predominance, and is characterized by overexpression of the ALK protein because of ALK gene translocation. Most patients with systemic ALCL present with advanced stage III or IV, which is frequently associated with systemic symptoms and extranodal involvement. The systemic symptoms in patients with ALK+ ALCL include weight loss, fever, weakness, fatigue, and night sweats. Common extranodal involvement includes involvement of the skin, bone, soft tissues, lungs, and liver (1).

Compared to other peripheral T-Cell lymphoma (PTCL) subtypes, ALK+ ALCL has a significantly better prognosis after first-line treatment. Following initial treatment, patients with ALK+ ALCL demonstrate relatively favorable outcomes, with complete remission (CR) rates of up to 86% and 8-year overall survival (OS) rates of 82% in a long-term follow-up study (2). However, relapsed and refractory ALK+ ALCL is associated with a relatively poor prognosis, and established standards for the management of relapsed or refractory disease are lacking. Currently, stem cell transplantation, targeted therapy, and immunotherapy with ALK inhibitors, brentuximab vedotin (BV), histone deacetylase (HDAC) inhibitors, and programmed cell death protein 1 (PD-1)/programmed death-ligand 1 (PD-L1) inhibitors are some of the most widely considered options for the treatment of relapsed or refractory ALK+ ALCL.

To date, consensus regarding the roles of autologous stem cell transplantation (ASCT) and CAR-T cell therapy in patients with relapsed/refractory ALK+ ALCL has not been achieved. In this study, we present the case of a patient who failed multiple lines of therapy and benefited from ASCT and CAR-T cell treatment. Our study provides insights into therapeutic strategies for such patients.

## 2 Case report

**Figure 1:**
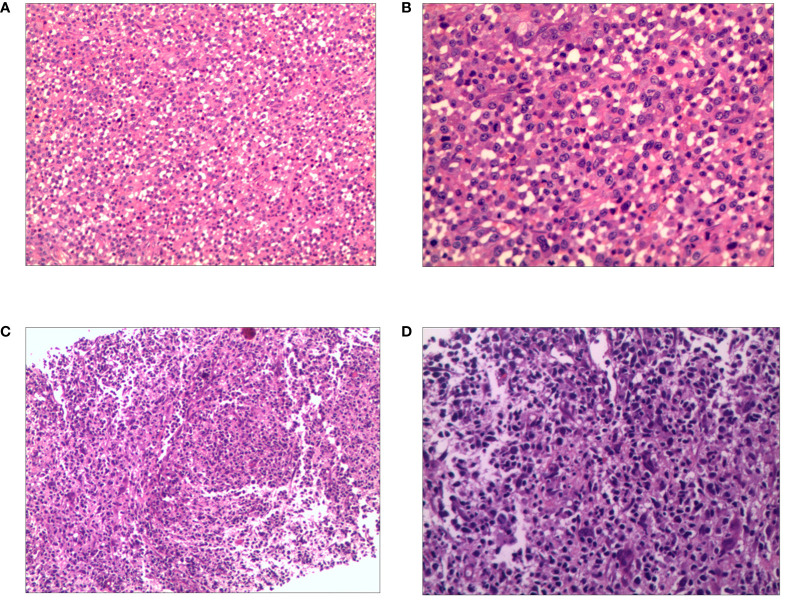
Hematoxylin and eosin (H&E) of relapsed/refractory anaplastic large cell lymphoma. The first biopsy: **(A)** Pathological HE image of the patient’s right axillary lymph node (100×). **(B)** Pathological HE image of the patient’s right axillary lymph node (200×). The second biopsy: **(C)** Pathological HE image of the patient’s abdomen mass (100×). **(D)** Pathological HE image of the patient’s abdomen mass (200×).

**Figure 2:**
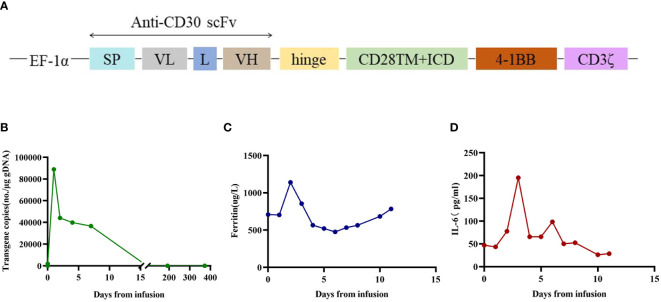
The therapeutic response after anti-CD30 CAR T-cell infusion. **(A)** Schematic diagrams of CAR construct. The third-generation CAR was composed of a single chain variable fragment (scFv), two costimulatory domains from CD28 and 4-1BB, and CD3ζ chain as activation domain. The scFv was derived from a murine monoclonal antibody against human CD30. Abbreviations: SP, signal peptide; VL, variable L chain; L, linker; VH, variable H chain. **(B)** The copies of CAR30 transgenes in the peripheral blood detected by ddPCR. **(C)** Dynamic changes in ferritin after CAR T cell infusion. **(D)** Dynamic changes in IL-6 after CAR T cell infusion.

**Figure 3:**
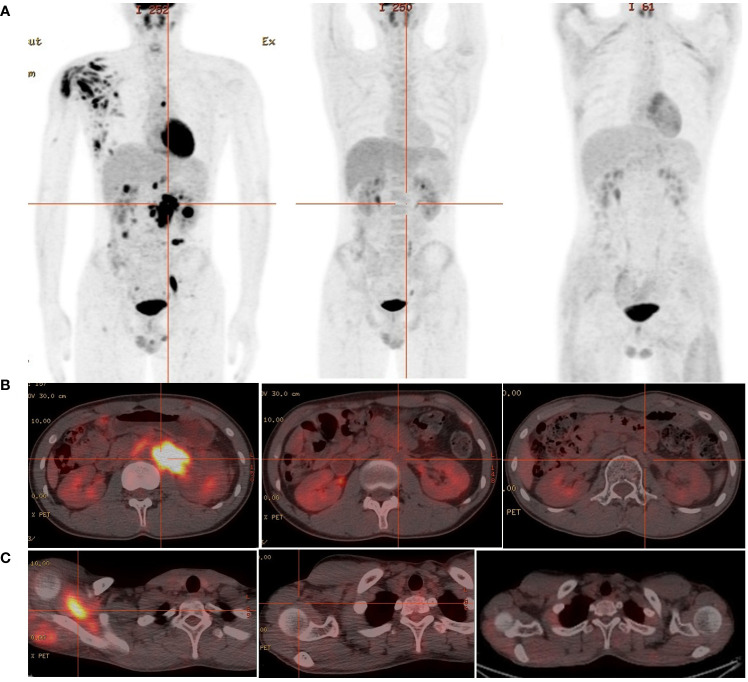
The 18F-FDG PET/CT images of relapsed/refractory ALCL. **(A)** From left to right: diagnosed as relapsed/refractory ALCL;1 month before ASCT and CAR T-cell therapy;3 months after ASCT and CAR T-cell therapy. **(B)** The metabolic images of retroperitoneal enlarged lymph nodes during disease recurrence, before ASCT and CAR T-cell treatment and 3 months after treatment (from left to right). **(C)** The metabolic images of right scapular muscle group during disease recurrence, before ASCT and CAR T-cell treatment and 3 months after treatment (from left to right).

**Figure 4:**
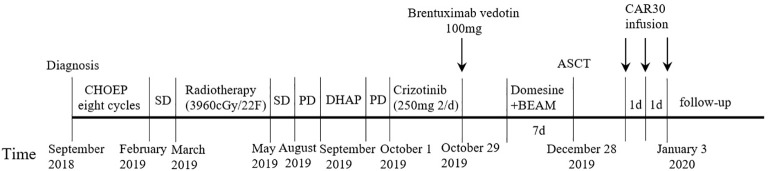
The timeline of disease status and the corresponding clinical treatment.

A 19-year-old man presented to our otolaryngology department in August 2017 with a table tennis-sized lump in his left chest wall and right axilla and pain in right rib that lasted for 1 month. The patient’s history was free of relevant diseases. The patient’s family history and genetic history are free of genetically related diseases and free of hematologic disorders such as lymphoma. Chest computed tomography (CT) showed enlargement of the right axillary lymph nodes, indicating neoplastic lesions and destruction of the right eighth rib bone. The diagnosis of ALCL was confirmed using a right axillary lymph node biopsy and immunohistochemical analysis (**Figure 1**). Immunohistochemical staining revealed that the tumor cells were positive for ALK and CD30. The Ki-67 proliferation index was 90%. The patient was diagnosed with stage IV ALCL and had an international prognostic index (PI) score of 3. The patient received induction chemotherapy, including 8 courses of cyclophosphamide, doxorubicin, vincristine, etoposide, and prednisolone (CHOEP). The patient underwent clinical evaluation after completing 8 cycles of chemotherapy. Positron emission tomography–computed tomography (PET–CT) revealed bilateral neck and axillary lymph node enlargement and increased metabolism. Subsequently, the patient underwent neck and axillary sensitization radiotherapy (Dt 3960cGy/22F). Three months later, a substantial subcutaneous mass measuring approximately 1 cm was palpated in the left lower abdomen. The patient was diagnosed with relapsed/refractory ALCL after an abdominal mass biopsy (**Figure 1**). PET–CT (August 29, 2019) demonstrated multiple mediastinal, abdominal, and retroperitoneal lymph node enlargement that were partially fused. The largest one was approximately 4.9 ×3.3 cm, and the SUVmax was 19.6. Right pleural thickening, a slightly low-density shadow in the left kidney, multiple muscles, and subcutaneously increased metabolism were also observed. These new changes were considered lymphoma infiltration. The bilateral cervical and axillary lymph nodes increased in size, and metabolism was enhanced. Increased local metabolism of the stomach, small intestine, and transverse colon was also observed. One cycle of dexamethasone, high-dose cytosine arabinoside, cisplatin (DHAP) chemotherapy was initiated on September 4, 2019. The patient presented with abdominal pain on September 23, 2019. Abdominal imaging revealed a significant increase and enlargement of the peripancreatic, retroperitoneal, and mesenteric root lymph nodes, indicating tumor progression. New flaky low-density shadows appeared in the left kidney, indicating a potential tumor invasion. Imaging assessment of the retroperitoneal mass showed that the stable disease (SD) developed into progressive disease (PD). The patient began taking crizotinib (250 mg twice daily) on October 1, 2019, for 1 month. BV at a dose of 100 mg was administered to the patient on October 29, 2019. On the day of BV infusion, the patient underwent an abdominal CT examination. Compared with the previous abdominal images (September 25, 2019), the number of enlarged retroperitoneal and mesenteric lymph nodes was significantly reduced. The range of patchy, low-density lesions in the left kidney also decreased significantly. Abdominal imaging findings were evaluated as partial remission (PR). The patient learned of the clinical trial of tandem ASCT and CAR30 T cell infusion in r/r CD30 + lymphoma being conducted at our hospital, to further enhance the curative effect, he volunteered to participate in the clinical trial. The trial was approved by the Institutional Review Board of Tongji Hospital, Tongji Medical College, Huazhong University of Science and Technology, and the study was registered with the Chinese Clinical Trial Registry (ChiCTR, number ChiCTR2100053662). Informed consent was obtained by the patient and her family according to the Declaration of Helsinki. On December 21, doxorubicin hydrochloride liposome with the BEAM regimen pretreatment was performed. Autologous hematopoietic stem cells (4.94 × 10^6^/kg CD34+ cells) were infused on December 28, and CD30 (24.00 × 10^6^/kg) CAR-T cells were infused three times. The three infusion doses of CD30 CAR-T were 5 × 10^6^/kg, 5 × 10^6^/kg and 1.4 × 10^7^/kg respectively (January 1, 2, and 3, 2020). The structure is as shown in **Figure 2A**. CAR30 transgene copy numbers in the peripheral blood was detected by droplet digital polymerase chain reaction (ddPCR) (**Figure 2B**). After infusion of anti-CD30 CAR T-cells, grade 1 cytokine release syndrome (CRS) was observed, and immune effector cell-associated neurotoxicity syndrome (ICANS) was not occur. The serum ferritin and interleukin-6 levels were assessed after cell infusion (**Figure 2**). The engraftment times of neutrophil and platelet after hematopoietic stem cells infusion were both 14 days. The results of the PET–CT evaluation 3 months after treatment showed that the retroperitoneal soft tissue focus was significantly reduced or had disappeared (**Figure 3**). PET–CT assessments at 3, 6, and 12 months after autologous stem-cell transplantation (ASCT) and CAR T-cell therapy showed sustained complete remission (CR). The latest PET–CT showed that the size of the lymph nodes in the left neck IIA region was similar, and metabolism was slightly reduced compared to previous PET–CT images (July 16, 2020). Approximately 4 years after ASCT and CAR T-cell therapy, the patient was disease-free. The timeline of clinical treatment and disease status is shown in **Figure 4**.

## 3 Discussion

It’s been a long time the recommended first-line treatment options for patients with ALCL are mostly anthracycline-based cyclophosphamide, vincristine, doxorubicin, and prednisone (CHOP) or CHOP-like regimens. CHOEP is more suitable for patients aged <60 years. In a previous study, among younger patients with ALK+ ALCL (n = 78) with normal LDH levels at the time of diagnosis, adding etoposide to CHOP (-like) regimens enhanced overall response rates and resulted in superior event-free survival (EFS) (3-year EFS of 91% vs. 57% in patients treated with CHOEP vs. CHOP, respectively). Furthermore, a large analysis of 263 adult patients with ALK+ ALCL demonstrated that the integration of etoposide into primary therapy was associated with significant improvements in the 5-year progression-free survival (PFS) (83% vs. 62%) and OS (93% vs. 74%) vs. non-etoposide regimens. In patients aged ≤60 years (n = 232), the respective 5-year PFS and OS were 81% vs. 65% and 92% vs. 77%, respectively (3). In Netherlands, a nationwide population-based study assessed the impact of etoposide on overall survival (OS) among patients aged 18 to 64 years with stage II to IV ALCL, angioimmunoblastic T-cell lymphoma (AITL), or PTCL not otherwise specified (NOS) diagnosed between 1989 and 2018. In patients with ALK+ ALCL who received CHOEP, CR rate was significantly higher than in patients who received CHOP (86% vs 61%). Overall, 5-year OS for patients with ALK+ ALCL who received CHOEP was superior to that in patients who received CHOP (90% vs 61%) (4). Multiple studies have shown that etoposide being of great value in ALCL but also in other PTCL subtypes. CD30 is a transmembrane glycoprotein receptor expressed on all systemic ALCL, making it an ideal therapeutic target. BV is a chimeric monoclonal antibody–drug conjugate that targets CD30. The phase 3 ECHELON-2 study comparing CHOP with BV (BV substituted for vincristine; BV–CHP) regimen to CHOP in CD30+ adult PTCLs, including ALK+ ALCL with IPI ≥2, showed an improved 3-year PFS (57.1% vs. 44.4%) and OS (76.8% vs. 69.1%) in the BV group (5). The ECHELON-2 trial established the BV–CHP regimen as a new standard front-line therapy for patients with ALK+ ALCL. After 5 years of follow-up, patients with PTCL treated with BV–CHP as a frontline treatment had a survival benefit over CHOP, with a 5-year OS of 70.1% vs. 61.0%, respectively. This study further demonstrated that BV–CHP resulted in clinically significant improvements in OS compared with CHOP (6). Children’s Oncology Group trial ANHL12P1 described the results of adding BV to standard chemotherapy in children with newly diagnosed ALK+ ALCL, with a 2-year EFS of 79.1% and OS of 97% (7). The addition of BV prevented relapses during therapy, and the OS and EFS estimates were relatively favorable to the results obtained using conventional chemotherapy. Thus, at present, BV-CHP (and its variations such as BV-CHEP) is the current standard of care for patients with ALCL (either ALK+ or ALK-) in the Europe and America.

Relapsed and refractory ALK+ ALCL are associated with a poor prognosis. NCCN guidelines recommend intensified chemotherapy with or without ASCT consolidation for r/r ALCL, however, this not effective treatment in all ALK+ALCL. Various therapeutic approaches, including high-dose chemotherapy regimens, new drugs such as anti-CD30 antibody drugs and ALK inhibitors, as well as autologous or allogeneic hematopoietic stem cell transplantation, have been used. ALK inhibitors are a more recently approved therapy that is often used as a stand-alone therapy for relapsed or refractory disease. Crizotinib, an inhibitor of ALK, was approved for the treatment of refractory ALK+ ALCL in pediatric patients and young adults in January 2021 (8).A small single-center study that evaluated crizotinib as a monotherapy for 25 patients with relapsed or refractory ALK+ ALCL found durable remission in almost 2/3 of patients (9). A non-controlled, open-label phase II study conducted in France enrolled 28 patients with relapsed/refractory ALK+ ALCL, of whom 25 patients receiving at least one dose of crizotinib were included in the analysis. The overall response rate at 8 weeks was 67% (95% CI: 47–82%), with 80% (95% CI: 44−97%) in children/adolescents and 57% (95% CI: 29−82%) in adults. The PFS and OS rates at 3 years were 40% (95% CI, 23–59%) and 63% (95% CI, 43–79%), respectively (10). In an open-label phase II trial, crizotinib was administered to 26 pediatric patients with relapsed or refractory ALK+ ALCL, achieving an objective response rate of 90% (11). Even though it induces CR in most cases, crizotinib has not yet been proven curative, as it may require life-long treatment. Notably, abrupt relapses of ALK+ lymphoma following crizotinib discontinuation have been reported. Crizotinib induces CR as a bridge for subsequent transplantation. Ceritinib, another ALK inhibitor, has shown a promising response as a treatment for ALK+ ALCL. The Phase I ASCEND-1 trial showed persistent responses in several adult patients with ALK+ ALCL, including three patients with relapsed ALK+ ALCL (12). Alectinib is a second-generation oral kinase inhibitor of ALK. A single-arm phase II study published in 2020 tested the efficacy of alectinib in 10 patients with relapsed/refractory ALK+ ALCL. Among these patients, eight (80%) achieved objective remission, and six (60%) achieved CR (13).

BV was approved in the USA and Europe for the treatment of relapsed ALCL in adults following the failure of at least one multi-agent chemotherapy protocol. BV has demonstrated its efficacy as a single agent (1.8 mg/kg every 3 weeks) in pediatric patients with relapsed ALCL, with objective response rates of 53–86% in phase 1 and 2 settings. A long-term follow-up of 5 years demonstrated that among a subset of patients with relapsed or refractory systemic ALCL, BV may be a potentially curative treatment option. Among all enrolled patients (n = 58), 16 (28%) had ALK+ ALCL, 10 achieved CR, and the PFS rate at 5 years was 50% among patients with ALK+ CR (95% CI, 19–81%) (14, 15). A multicenter study conducted in Italy evaluated the effectiveness of BV in 40 patients with relapsed/refractory ALCL, including 18 patients with ALK+. A total of 31 (77.5%) patients achieved a favorable response after a median of four cycles of BV monotherapy, with an overall response rate of 62.5% (16). Other immunotherapies and targeted therapies, such as PD-1/PD-L1 and HDAC inhibitors, can also be applied in ALCL. The use of PD-1/PD-L1 inhibitors in relapsed/refractory ALK+ ALCL has been demonstrated in previous case reports. Two patients with ALK + ALCL who relapsed after multiline therapy achieved sustained CR following nivolumab (3 mg/kg/2 for 2 weeks) (17, 18). Some non-randomized single-arm trials and small patient population studies have investigated the role of HDAC inhibitors, such as chidamide, romidepsin, and belinostat, combined with chemotherapy, in the first-line treatment of patients with ALCL (19–22).

Autologous hematopoietic stem cell transplantation (auto-HCT) following high-dose chemotherapy remains an option for the treatment of relapsed/refractory ALK+ ALCL and is associated with a 5-year PFS rate of up to 56% (23). A prospective cohort study demonstrated that high-dose chemotherapy followed by ASCT achieved a remarkable long-term complete response, with a 12-year overall survival rate of 62% in patients with ALK+ ALCL (24). In a single-arm, open-label, multicenter, phase 2 study, 16 patients with relapsed/refractory ALCL who responded to BV and achieved CR and subsequently received either allogeneic or autologous consolidative SCT had a 5-year PFS rate of 69% (95% CI, 46–91%) and OS rate of 75% (95% CI, 54–96%) (14). A large cohort study performed by the Center for International Blood and Marrow Transplant Research found that patients with relapsed ALCL undergoing auto-HCT had superior outcomes to those receiving allo-HCT, with a smaller non-relapse mortality at 100 days, 1 year, and 3 years, and superior PFS and OS at 1 and 3 years for the patients who underwent auto-HCT compared with those who underwent allo-HCT (23). CAR-T therapy represents a major breakthrough in relapsed/refractory B cell NHL immunology. However, its application in T-cell malignancies is still being explored due to issues such as antigen targetability and cell fratricide. The presence of CAR-T cells that can recognize CD30 offers hope for patients with ALCL. Clinical trials of CD30-directed CAR-T cells for relapsed/refractory CD30+ ALCL are currently ongoing (NCT04653649, NCT04008394, NCT04288726, and NCT04288726). An open-label, single-center, single-arm pilot study demonstrated the combined administration of ASCT and CAR30 T-cell therapy was well-tolerate and highly effective in r/r classical Hodgkin lymphoma (cHL) and ALCL. In six r/r CD30+ lymphoma patients (five with cHL and one with ALK-negative ALCL), ORR and CR rates were 100% and 83.3%, respectively (25). Given the limited data on ASCT and sequential anti-CD30 CAR T cell therapy, it is difficult to know if the CAR-T cell therapy added any improved survival over the ASCT alone.

In the present case, the disease recurred quickly after the patient received chemotherapy and local radiotherapy. The use of crizotinib and BV as a bridge to autologous stem cell transplantation followed by CD30-directed chimeric antigen receptor T cell therapy is expected to achieve a long-term survival effect in the management of patients with relapsed/refractory CD30+ ALK+ ALCL. For patients with ALK+ ALCL with early relapse or refractory disease, targeted therapy induced remission may be considered as a bridge to transplantation or subsequent ASCT combined with CAR30 T-cell therapy as a treatment option. This is a promising approach for future clinical trials. Further prospective studies with larger sample sizes are required to demonstrate the superiority of the therapeutic strategies mentioned in this study for relapsed/refractory ALK+ ALCL.

## 4 Patient perspective

When discussing the feelings from diagnosis to recovery, the patient felt that everything was as surreal as a dream. His life went through significant ups and downs but turned out well. The patient’s successful recovery was attributed to the combined efforts of the doctors and the wholehearted dedication and support of his parents. At present, the patient has returned to campus in good physical condition, with gratitude, optimism, and hope for the future.

## Data availability statement

The original contributions presented in the study are included in the article/supplementary material. Further inquiries can be directed to the corresponding authors.

## Ethics statement

Written informed consent was obtained from the individual(s) for the publication of any potentially identifiable images or data included in this article. Written informed consent was obtained from the participant/patient(s) for the publication of this case report.

## Author contributions

WL: Writing – original draft. JW: Data curation, Writing – original draft. XM: Data curation, Writing – original draft. QZ: Formal analysis, Writing – original draft. DZ: Formal analysis, Writing – original draft. RZ: Data curation, Writing – original draft. MZ: Writing – original draft. ZS: Writing – original draft. LC: Writing – review & editing. XZ: Writing – review & editing. YX: Funding acquisition, Writing – review & editing.
